# Revision of the Palaearctic species of *Lysitermus* Foerster (Hymenoptera, Braconidae, Hormiinae)

**DOI:** 10.3897/zookeys.1040.66274

**Published:** 2021-05-28

**Authors:** Cornelis van Achterberg, Fredrik Skeppstedt, Simo Väänänen

**Affiliations:** 1 Naturalis Biodiversity Center, Postbus 9517, 2300 RA Leiden, Netherlands Naturalis Biodiversity Center Leiden Netherlands; 2 Tegelslagarevägen 44, 29144 Kristianstad, Sweden Unaffiliated Kristianstad Sweden; 3 Ojahaanpolku 6 E 64, 01600 Vantaa, Finland Unaffiliated Vantaa Finland

**Keywords:** Bulgaria, *Diplodoma laichartingella*, Finland, France, key, Lysitermini, Malta, new record, Portugal, Psychidae, Romania, Sweden

## Abstract

The three Palaearctic species of *Lysitermus* Foerster, 1863 (Braconidae, Hormiinae, Lysitermini) are revised. The type species is described for the first time together with both of the other species. *Lysitermus suecicus* (Hedqvist, 1957) is a new synonym of *L. tritoma* (Bouček, 1956), and *L. longiventris* (Tobias, 1976) of *L. talitzkii* (Tobias, 1971), **stat. nov.**

## Introduction

The little-known Palaearctic and Afrotropical genus *Lysitermus* Foerster, 1863 (Braconidae, Hormiinae, Lysitermini) was described by Foerster in 1863 without a description of its type species, *L. pallidus* Foerster, 1863. The identity of the genus was unclear, which resulted in three new generic names for this taxon (*Rogadinaspis* Bouček, 1956; *Paracedria* Hedqvist, 1957; *Prolysitermus* Tobias, 1971). Hedqvist (1963) recognised his earlier mistake and synonymised the first two with *Lysitermus* Foerster. He also synonymised their type species (*Rogadinaspis tritoma* Bouček, 1956 and *Paracedria suecicus* Hedqvist, 1957) with *L. pallidus*. The type series of *Lysitermus* was examined by the first author in 1979, and it proved to be also congeneric with *Prolysitermus* Tobias (van Achterberg 1982) and *Lysitermus* sensu Tobias (1971, 1976) was renamed in *Tritermus* van Achterberg, 1982. In the generic revision of Afrotropical and West Palaearctic genera of Rogadinae (van Achterberg 1991) the author attempted to construct the first key to the European species based mainly on the type series and their original descriptions (but the latter was absent for *L. pallidus*). Four species were recognised by using the shape of the second metasomal suture (sinuate in *L. tritoma*; Figs 24, 25), subparallel-sided metasoma (of *L. longiventris* (Tobias, 1976)) and the relative length of the median carina of the propodeum (for *L. pallidus* and *L. suecicus*). *Lysitermus talitzkii* (Tobias, 1971) was synonymised with *L. pallidus* by Belokobylskij and Tobias (1986).

The second author reared a series of *L. pallidus* from *Diplodoma laichartingella* (Goeze, 1783) (Lepidoptera, Psychidae). After studying the reared material, plus small reared series in the National Museums of Scotland and Naturalis Biodiversity Center, it turned out that there are only two species present in the material from western Europe, with most likely a third one in south-eastern Europe, and that the characters used in the previous key (van Achterberg 1991) were too variable to be useful. In this paper, a new key is presented and *L. pallidus* is described and illustrated for the first time, together with a redescription of *L. tritoma* and *L. talitzkii*.

Although developmental details are hardly known, *Lysitermus* species have been reared as solitary or weakly gregarious parasitoids of case-bearing lepidopterous larvae of Psychidae (*Luffia* and *Diplodoma* spp.) and Tineidae (*Eudarcia derrai* (Gaedike, 1983)) (Gupta and Quicke 2018; Mifsud et al. 2019; this paper). Recently, one species has been found in a mass-rearing from *Inonotus radiatus* bracket fungus on common alder (*Alnus glutinosa* (L.)) in Sweden (Jonsell et al. 2016), probably from Psychidae or Tineidae hiding or feeding in the fungus.

## Material and methods

The specimens studied were (rarely) collected in Malaise traps and directly conserved in 70% alcohol or reared from their hosts and preserved dry. For identification of the subfamily Lysiterminae, see van Achterberg (1993), and for identification of the genus *Lysitermus*, see van Achterberg (1991).

Morphological terminology follows van Achterberg (1988, 1993), including abbreviations for wing venation. Measurements are taken as indicated by van Achterberg (1988): for the length and the width of a body part the maximum length and width is taken, unless otherwise indicated. The length of the mesosoma is measured from the anterior border of the mesoscutum to the posterior border of the propodeum and of the first tergite from the posterior border of the adductor to the medio-posterior margin of the tergite. An asterisk indicates a new record for the country.

Observations and descriptions were made with an Olympus SZ40 stereomicroscope with 2× objective lens and fluorescent lamp. Photographic images were made with Sony A7RIII 42.4MP camera combined with Canon MPE 65 mm/1–5× macro lens at f2.8 and a Youngnuo YN14EX ring flash. For photo stacking Helicon Focus 7 software (method C pyramid) was used. Additional photos of the Finnish specimen were made with a Nikon DS-Ri2 camera mounted on Nikon SMZ25 stereomicroscope and combined with Zerene Stacker focus stacking software. **BZL** stands for Oberösterreichisches Landesmuseum, Biologiezentrum, Linz; **CSV** for Simo Väänänen Collection; **FMNH** for Finnish Museum of Natural History, Helsinki; **MTMA** for Hungarian Natural History Museum, Budapest; **NMS** for National Museums of Scotland, Edinburgh; **NRS** for Swedish Natural History Museum, Stockholm; **RMNH** for Naturalis Biodiversity Center, Leiden; **ZJUH** for Zhejiang University, Hangzhou; **ZISP** for Zoological Institute of the Russian Academy of Sciences, Saint Petersburg, Russia and **ZMB** for Zoologisches Museum, Humboldt Universität, Berlin.

## Taxonomy

### Lysitermus

Taxon classificationAnimaliaHymenopteraBraconidae

Foerster, 1863

CF439742-C97F-5F30-9E7D-0F5A2E210B08

Figs 1 , 2–8 , 9–11 , 12 , 13–18 , 19–21 , 22–25 , 26–27 , 28–29 , 30–32 , 33 , 34–42 , 43–44 , 45–47

Lysitermus Foerster, 1863: 236 [not Tobias 1971: 205, 1976: 49]; Hedqvist 1963: 35; Shenefelt 1975: 1154–1155; van Achterberg 1982: 125; Belokobylskij and Tobias 1986: 63–64; van Achterberg 1991: 19, 1995: 93; Jonsell et al. 2016: 12, 18; Forshage et al. 2016: 12. Type species (by monotypy): Lysitermus pallidus Foerster, 1863 [examined].
Rogadinaspis Bouček, 1956: 441; Hedqvist 1963: 35 (as synonym of Lysitermus Foerster, 1863); Shenefelt 1975: 1155; van Achterberg 1991: 19, 1995: 93. Type species (by monotypy): Rogadinaspis tritoma Bouček, 1956 [examined].
Paracedria Hedqvist, 1957: 219; Hedqvist 1963: 35 (as synonym of Lysitermus Foerster, 1863); Shenefelt 1975: 1155; van Achterberg 1991: 19, 1995: 93. Type species (by monotypy): Paracedria suecicus Hedqvist, 1957 [examined].
Prolysitermus Tobias, 1971: 205–206; Shenefelt 1975: 1155; Tobias 1976: 49; van Achterberg 1982: 125 (as synonym of Lysitermus Foerster, 1863), 1991: 19, 1995: 93. Type species (by monotypy): Prolysitermus talitzkii Tobias, 1971 [examined].

#### Diagnosis.

See van Achterberg (1991: 19). The type species of *Lysitermus* Foerster was not described in 1863; the species name is valid because it was the only species included in the new genus. Indirectly, the type species is characterised by the two features mentioned in the key to the genera (vein 2-SR of fore wing absent and only three metasomal segments visible) but its unequivocal recognition has been problematical. Therefore, we illustrate here recently reared specimens from Sweden, because the cotypes of *L. pallidus* in ZMB are less suitable for redescription and in any case not currently available.

**Figure 1.**
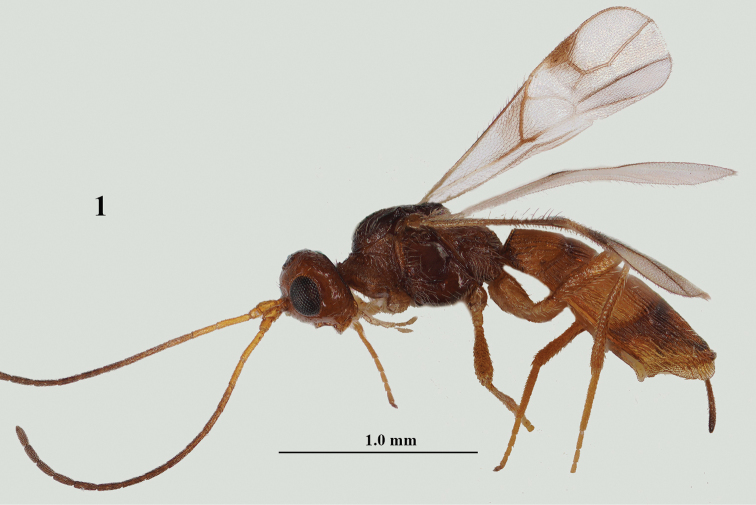
*Lysitermus pallidus* Foerster, ♀, Sweden, habitus lateral. Photograph: R. Soethof.

#### Biology.

Facultative gregarious parasitoids of case-bearing lepidopterous larvae of Psychidae and Tineidae; they are almost certainly idiobiont ectoparasitoids (Gupta and Quicke 2018; Mifsud et al. 2019; this paper). There is no mummification of the host larva, as in Rogadinae, and the host remains in the host case are compatible with ectoparasitoism (M.R. Shaw pers. comm.). The records of Scolytini (*Pityophthorus micrographus* (Linnaeus, 1758), *Polygraphus poligraphus* (Linnaeus, 1758)) are based on a mass rearing from a dead *Picea abies* tree infested by both species (Hedqvist 1957). As *Lysitermus* has been reared from case-bearing larvae so far and there is no direct rearing known from Curculionidae (Scolytini), we consider both records very doubtful.

#### Notes.

**Figures 2–8.**
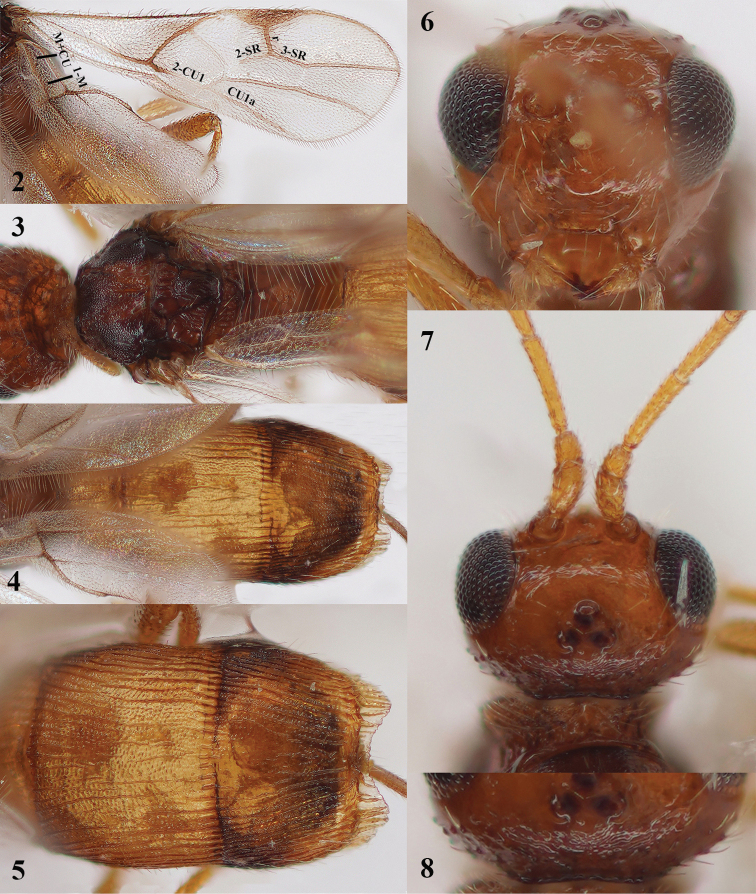
*Lysitermus pallidus* Foerster, ♀, Sweden **2** wings **3** mesosoma dorsal **4** metasoma dorsal **5** second and third metasomal tergites dorsal **6** head anterior **7** head dorsal **8** detail vertex dorsal. Photographs: R. Soethof.

*Lysitermus* Foerster and the widespread Old World genus *Acanthormius* Ashmead, 1906, are very similar and should be synonymised in future if molecular data show that *Acanthormius* and *Lysitermus* are paraphyletic. Up to now, with only few species sampled, the Old World *Lysitermus* species sampled clusters with *Afrotritermus* Belokobylskij, 1995 and *Atritermus* Belokobylskij, Zaldivar-Riverón & Quicke, 2007 and not with the *Acanthormius* clade (Jasso-Martínez et al. 2021). Therefore, we refrain from synonymising both genera, despite that both have vein CU1a of the fore wing at the level of vein 2-CU1 or above (Fig. 2) and lack a parastigma (but the parastigma is also rarely absent in *Aulosaphoides* van Achterberg, 1995). However, in *Acanthormius* vein 2-SR of the fore wing is complete and the third tergite, excluding lamella, usually protruding latero-apically. In *Lysitermus* vein 2-SR is often largely or entirely absent, if present then nearly always its posterior third unpigmented and only the lamella of the third tergite is protruding. The development of vein 2-SR is very variable; the specimen in Figure 2 has the vein in one wing only pigmented, as figured, but in the other wing entirely sclerotised. In *Aulosaphoides* vein CU1a of the fore wing is situated distinctly below the level of vein 2-CU1 (at the same level or above in *Lysitermus*) and vein r is emitted distinctly before the middle of the pterostigma (submedially in *Lysitermus*). *Trissarthrum* Ashmead, 1900, is traditionally included in *Lysitermus* (Wharton 1993; van Achterberg 1995; Yu et al. 2016), but its Neotropical type species, *T. maculipennis* Ashmead, 1900, from St. Vincent has a complete vein 2-SR of the fore wing and the propodeal areola comparatively narrow. Therefore, it may be a different Neotropical genus near *Acanthormius* lacking the apico-lateral protruding part of the third tergite and having vein M+CU1 of the fore wing non-tubular and vein 1-M of hind wing much longer than vein M+CU (van Achterberg 1995). Both characters are also present in the only other described species from the New World, *L. woolleyi* Wharton, 1993 from Mexico. The Australian *Lysitermus* sp. 1 listed by Jasso-Martínez et al. (2021) clusters with the Afrotropical genera *Afrotritermus* Belokobylskij, 1995 and *Atritermus* Belokobylskij, Zaldívar-Riverón & Quicke, 2007. The listed Neotropical *Lysitermus* sp. 2 (= *Trissarthrum*) clusters with the *Acanthormius* clade (Jasso-Martínez et al. 2021). *Lysitermus* without *Trissarthrum* has an Old World distribution, known from the Afrotropical (Papp and van Achterberg 1999; van Achterberg 2000), Australian (Jasso-Martínez et al. 2021), and Palaearctic regions (Yu et al. 2016).

The position of the tribe Lysitermini is uncertain, but there is increasing evidence for a subordinate position in the Hormiinae. Recently, Lysitermini are either included as a tribe in the Rogadinae*sensu lato* (Chen and van Achterberg 2019) or the Hormiinae (Jasso-Martínez et al. 2021), or treated as a separate subfamily (Quicke et al. 2020).

**Figures 9–11.**
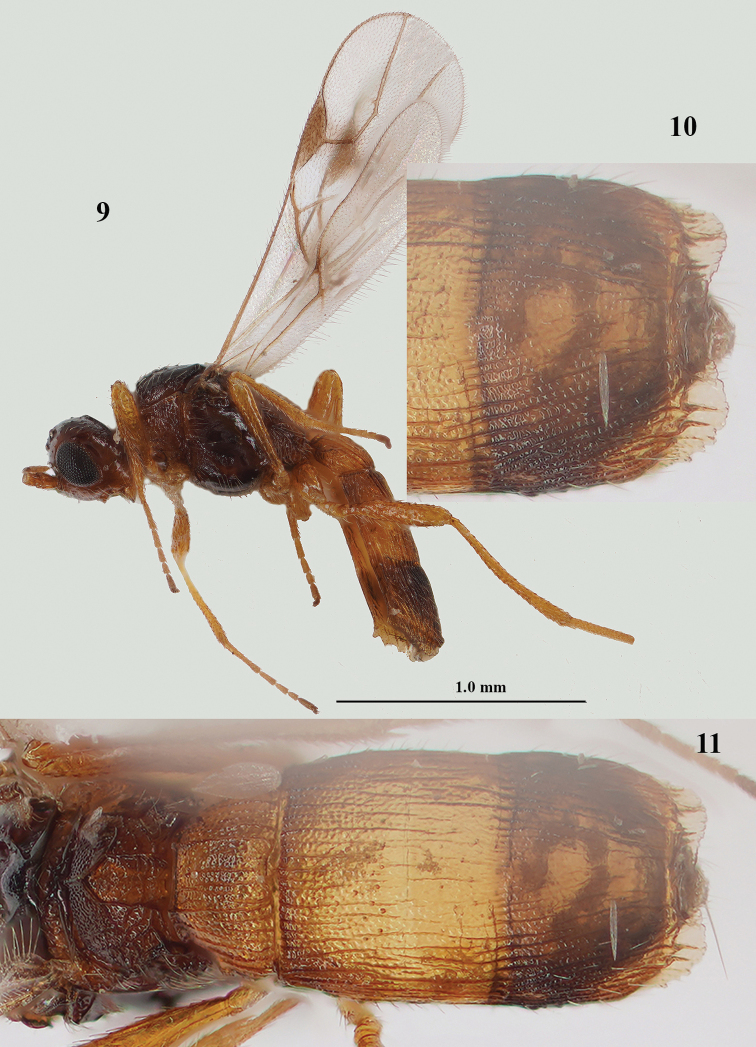
*Lysitermus pallidus* Foerster, ♂, Sweden **9** habitus lateral **10** third metasomal tergite dorsal **11** propodeum and metasoma dorsal. Photographs: R. Soethof.

##### Key to Palaearctic species of the genus *Lysitermus* Foerster

**Table:** 

1	Area behind stemmaticum finely granulate and more or less rugulose anteriorly (Fig. 8); scutellum finely striate antero-laterally and granulate medially (Fig. 3); apical lamella of third metasomal tergite in ♀ distinctly protruding laterally (Fig. 1), with approx. 25 carinae and wide in dorsal view (Figs 4, 5), its border distinctly serrate in lateral view (also in ♂ comparatively wide, but in lateral view less serrate, without smooth apical rim; Figs 9–11); third tergite of ♀ mainly yellowish brown, less contrasting with second tergite (Figs 1, 5, 26; more or less darkened in ♂: Fig. 9)	***L. pallidus* Foerster, 1863**
–	Area behind stemmaticum smooth or largely so (Figs 18, 32, 41); scutellum smooth, without lateral striae (Figs 14, 28, 32, 36, 41), but rarely granulate medially; apical lamella of third tergite less protruding laterally (Figs 12, 19, 23, 29, 30, 39), with 8–16 carinae and medium-sized in dorsal view (Figs 16, 21, 24, 25, 27, 31), usually straight to slightly serrate in lateral view (Figs 12, 29, 30, 39); third tergite dark brown or brown, darker than yellowish brown second tergite (Figs 15, 20, 22, 31), but in melanistic specimens second tergite more or less dark brown (Figs 27–29, 39, 43)	**2**
2	Apical lamella of third metasomal tergite concave medio-posteriorly (Figs 16, 21, 24), but sometimes intermediate (Figs 25, 27) and with a distinct and smooth rim in front of it (Figs 15, 20, 23, 27); hind tibia (except apically) parallel-sided or nearly so (Figs 12, 29); third antennal segment yellow or yellowish brown (Figs 12, 17, 19, 22, 29), rarely mainly dark brown; vein 3-SR of fore wing 1.3–2.0× longer than vein r (Figs 13, 22)	***L. tritoma* (Bouček, 1956)**
–	Apical lamella of third metasomal tergite straight medio-posteriorly or nearly so (Figs 31, 37, 39, 44, 47) and rim in front of it less developed and more or less sculptured (Figs 30, 31, 33, 37, 46, 47); hind tibia gradually widened (Figs 30, 45); third antennal segment dark brown or largely so (Figs 33, 45); vein 3-SR of fore wing usually 2.3–3.0× longer than vein r (Figs 30, 33, 34), but sometimes approx. 1.4× (Figs 43, 45)	***L. talitzkii* (Tobias, 1971), stat. nov.**

### Lysitermus pallidus

Taxon classificationAnimaliaHymenopteraBraconidae

Foerster, 1863

D3A25B4C-669B-5988-99DD-B3B344CA857E

Figs 1 , 2–8 , 9–11 , 26

Lysitermus pallidus Foerster, 1863: 236; Shenefelt 1975: 1155; Belokobylskij and Tobias 1986: 64; van Achterberg 1991: 20; Gupta and Quicke 2018: 429.

#### Material.

5 ♀ + 1 ♂ (= type series of *L. pallidus*; ZMB), “[**Germany**], Aachen”, “Frst”, “*pallidus* Furst”, “Fam. 3 Hecaboloidae, n. gen. (rest illegible)”; 4 ♀ + 1 ♂ (RMNH), “**Sweden**: Skåne, Fjälkestad, Balsberget, ex cases of *Diplodoma laichartingella* (Goeze) (Psychidae), coll. 25.vi.2019, em. (indoors) 18.ii.2020, F. Skeppstedt”; 1 ♀ (NMS), “**Finland**: Lemland, Flaka, Björkö, 59.98°N, 20.19°E AI, 3.viii.2004, ex ?Psychid [= *Diplodoma laichartingella*] case, N. R. Fritzen”, “♀ Ant 17, *Lysitermus pallidus* Foerster, det. M. R. Shaw 2015”.

#### Redescription.

Figured and reared ♀ from Sweden (RMNH), length of body 2.2 mm, and of fore wing 1.6 mm.

***Head*.** Antenna 1.2× as long as fore wing, with 16 segments, slightly widened apically (Fig. 1), scapus oblique apically, length of third segment 1.3× fourth segment; third, fourth and penultimate segments 5.0, 4.0, and 2.4× their width, respectively; face largely smooth, with long erect setae and 1.7× wider than high; clypeus smooth, upper half distinctly convex and ventral half depressed; head in dorsal view 1.8× broader than long medially, eye 1.3× longer than temple dorsally, strongly and roundly narrowed posteriorly (Fig. 7); vertex finely granulate and superficially rugulose near stemmaticum (Fig. 8); occipital carina strong and in front of it crenulate; POL: diameter of posterior ocellus: OOL = 4:2:6; eye in lateral view 1.6 × as high as wide, temple distinctly broadening ventrally and width of eye 1.2× minimum width of temple (Fig. 1); malar space 0.7× as long as height of eye and 1.3× as long as basal width of mandible; frons smooth, except for some fine rugulae.

***Mesosoma*.** Mesosoma 1.4× as long as high in lateral view; mesoscutum granulate and with rather long, narrow, medio-posterior groove (Fig. 3); notauli shallow, complete, largely smooth and anteriorly connected to lateral irregular carina; scutellar sulcus with 3 carinae; scutellum finely striate antero-laterally and superficially granulate medially (Fig. 3); propodeum granulate and with large areola, its median carina much shorter than anterior side of areola (Fig. 3); side of pronotum rugose-crenulate and secondary granulate sculpture; mesopleuron largely smooth and convex (Fig. 1); precoxal sulcus rather deep and only anterior half present, with few crenulae; metapleuron densely rugulose and with long setae; mesosternal sulcus deep, narrow and smooth. ***Wings*.** Fore wing: pterostigma elongate triangular and 4× longer than wide (Fig. 2), r issued near its middle and 1.2× longer than width of pterostigma; 2-SR complete and sclerotized left wing) or only basal two-thirds pigmented (right wing, Fig. 2); 3-SR 1.6× longer than r; SR1 straight and reaching tip of wing; m-cu distinctly postfurcal, rather short (Fig. 2); subdiscal cell distally closed, CU1b close to m-cu and CU1a at same level as 2-CU1 (Figs 1, 2). ***Legs*.** Hind coxa largely smooth; length of femur, tibia and basitarsus of hind leg 5.5, 11.4, and 10.2× their width, respectively; hind tarsus compressed.

***Metasoma*.** Length of first tergite 0.6× its apical width, its surface longitudinally striate and with additional granulate sculpture between striae, dorsal surface evenly convex, its dorsal carinae lamelliform and medially interconnected; medial length of second tergite 0.9× its basal width, and 1.4× as long as third tergite; second and third tergites longitudinally striate (but on middle of third tergite weakly developed) and secondary granulate sculpture; second transverse suture coarsely crenulate and nearly straight (Fig. 4); third tergite antero-laterally without minute, tooth-shaped protuberance and distinctly narrowed posteriorly (Figs 4, 5), with narrow, rugulose, and rather dull apical rim medially (Fig. 5) and distinctly serrate latero-apically (Fig. 4); apical lamella of third metasomal tergite distinctly protruding laterally (Fig. 1), concave and wide in dorsal view, with approx. 25 carinae (Figs 4, 5); setose part of ovipositor sheath 0.23 × as long as fore wing and 0.5 × as long as hind tibia, nearly parallel-sided (Fig. 1).

***Colour*.** Yellowish brown; third tergite mainly yellowish brown but laterally darkened, not contrasting with similarly coloured second tergite (Figs 1, 5, 26); mesosoma brown, but mesoscutum and scutellum largely dark brown; antenna (basal segments yellowish), pterostigma (but basal quarter yellow) and veins M+CU1 apically, 1-CU1, 1-SR, 1-M, r, and veins of apical half of fore wing dark brown (Fig. 2); palpi and tarsi pale yellowish; remainder of legs yellowish brown; wing membrane infuscate, but with subhyaline band below base of pterostigma (Fig. 1).

***Variations*.** Antenna with 16–17 segments; length of body 2.1–2.3 mm, and of fore wing 1.6–1.7 mm; length of ovipositor sheath 0.21–0.23× as long as fore wing; vein 2-SR of fore wing varies in reared series from nearly complete to entirely absent (Fig. 2), most frequently only basal half present as pigmented but unsclerotised vein; notauli complete to posterior third obsolescent; second metasomal tergite 1.3–1.5× longer than second tergite; lamella of third tergite more or less serrate (Figs 1, 5).

**Male.** Very similar to female, but metasoma slenderer (Fig. 11); head dark brown dorsally; third tergite more or less darkened in ♂ (Fig. 9), striate and/or granulate and its lamella serrate and with approx. 20 carinae (Figs 9–11); pterostigma dark brown basally.

#### Distribution.

Finland, Germany, Moldova, *Sweden.

#### Biology.

Five specimens of *Lysitermus pallidus* hatched from ten final instar larval cases of *Diplodoma laichartingella* (Goeze, 1783) (Lepidoptera, Psychidae) collected in Sweden by the second author. Dissection of the final instar larval cases showed that only three had been parasitized and five specimens had hatched from them. It clearly indicates that *L. pallidus* is a gregarious larval ectoparasitoid of this host, but probably a facultative one perhaps depending on the size (first- or second-year stage?) of the host. In northern Europe *D. laichartingella* has a two-year life cycle which raises the question of whether *L. pallidus* is a parasitoid only of fully grown larva and, therefore, has a two-year lifecycle like its host (Kunz 1989) or whether it might be able to complete its lifecycle in a juvenile larval case as well. The *D. laichartingella* cases are usually found in woodland areas where the caterpillar feeds on algae/mosses as well as dead insects on tree trunks, especially, of *Fagus sylvatica*L. and *Quercus robur*L. The specimen from Finland has also been reared from *D. laichartingella* (det. M. Mutanen) but was referred by Gupta and Quicke (2018) as reared from an unidentified psychid host.

### Lysitermus tritoma

Taxon classificationAnimaliaHymenopteraBraconidae

(Bouček, 1956)

3DC6A9F0-C2F0-5CA2-8620-78310B4BBA8B

Figs 12 , 13–18 , 19–21 , 22–25 , 26–27 , 28–29

Rogadinaspis tritoma Bouček, 1956: 441.
Lysitermus tritoma ; Hedqvist, 1963: 35; Shenefelt 1975: 1155; van Achterberg 1991: 20; Gupta and Quicke 2018: 429; Mifsud et al. 2019: 54 [examined].
Paracedria suecicus Hedqvist, 1957: 219.
Lysitermus suecicus ; Hedqvist, 1963: 35 (as synonym of L. pallidus); Shenefelt 1975: 1155; van Achterberg 1991: 20; Gupta and Quicke 2018: 429; Mifsud et al. 2019: 54 [examined]. Syn. nov.

#### Material.

1 ♀ (FMNH), “**Finland**: Ab, Parainen, Pexor, 60.26°N, 22.25°E, Malaise trap 1a, 25.vi.–6.vii.2020, Juho Paukkunen”; 1 ♀ (CSV), same label data, but 6–19.vii.2020, Juho Paukkunen & Jonathan Scotson; 1 ♀ + 1 ♂ (RMNH), “S. **Sweden**: Uppland, Edsbro, Kristineholm, S15 or S20, coll. 28.i.2009, ex *Inonotus radiatus* on *Alnus glutinosa*, C. Gonzales Alonso, RMNH’11”; 1 ♂ (RMNH), “**Netherlands**: UT, UTM FF 6560, Amerongen, unmanaged *Quercus robur* [forest], ex dead stem [in] cage 9(h), 29.v.–12.vi.2001, L. Moraal, RMNH’02”; 1 ♀ (NMS), “**France**: Lot-et-Garonne, Bernac, 28.vii.[19]90, M.R. Shaw”; 1 ♀ + 2 ♂ (RMNH), “**Spain**, Llansa, 1986, ex *Luffia lapidella*, H. Hendrickx, RMNH’96”; 1 ♀ (RMNH), “**Portugal**, Oeiras, 18–22.viii.1979, A. van Harten”; 9 ♀ + 4 ♂ (RMNH), “Portugal, Cascais fort, ex *Luffia lapidella*, coll. 27.vii.1994, H. Hendrickx”; 5 ♀ + 3 ♂ (RMNH), idem, but coll. 4.v.1995, ex *Luffia* sp.; 1 ♀ (RMNH), idem, but coll. 1.iii.1995; 10 ♀ + 2 ♂ (RMNH, ZJUH), idem, but Cascais (and mislabelled as from Azores), ex *Luffia ferchaultella* (Psych.), coll. 27.vii.1994, em. 21.viii.1994; 2 ♀ (RMNH, MTMA) “**Italy**: Sardinia centr., Bruncu Istiddi, 900 m, ex *Luffia* sp. n.?, viii.1975, E. Hartig & Gozmany”; 1 ♀ + 1 ♂ (NMS), “**Malta**, Buscett, 22.xi.2006, ex larva on *Luffia lapidella* (Goeze, 1783) [Psychidae], M. Zerafa”; 2 ♀ + 1 ♂ (NMS), “Malta: Mosta valley, larva on *Eudarcia derrai* (Gaedike, 1983) [Tineidae], coll. 22.ii.2010, em. v.2010, M. Zerafa”; 3 ♀ + 5 ♂ (BZL, NMS), “**Romania**: Siebenbürgen, Munt Apuseni (Trascaului), Umg. Posaga 2 km oberhalb, 600 m, ex *Dahlica rakosy* oder *Apt. helicoidella* [Psychidae], em. Ende April 2005, M. Weidlich”; 2 ♀ + 1 ♂ (BZL, NMS), “**Bulgaria**: Macedonia, Pirin planina – Süd, Umg. Jane Sandanski, 1220 m, el. 13.v.2000, M. Weidlich, ex *Dahlica* sp. [Psychidae]”.

#### Redescription.

Figured ♀ from France (NMS), length of body 1.6 mm and of fore wing 1.4 mm.

**Figure 12.**
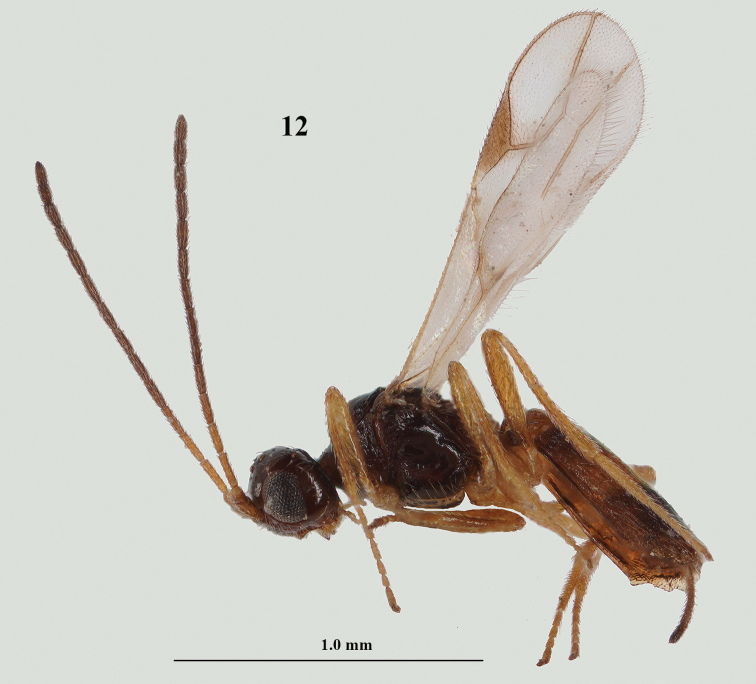
*Lysitermus tritoma* (Bouček), ♀, France, habitus lateral. Photograph: R. Soethof.

***Head*.** Antenna 1.1× as long as fore wing, with 15 segments, rather widened apically (Fig. 12), scapus oblique apically, length of third segment 1.1× fourth segment; third, fourth and penultimate segments 5.0, 4.5, and 3.1× their width, respectively; face largely smooth, with long erect setae and 1.7× wider than high; clypeus smooth, upper half distinctly convex and ventral half depressed; head in dorsal view 1.8× broader than long medially, eye 1.4× longer than temple dorsally, strongly and roundly narrowed posteriorly (Fig. 18); vertex smooth and shiny, including area near stemmaticum (Fig. 18); occipital carina moderately strong and in front of it indistinctly micro-crenulate; POL: diameter of posterior ocellus: OOL = 2:2:3; eye in lateral view 1.6× as high as wide, temple distinctly broadening ventrally, smooth and width of eye 1.6× minimum width of temple laterally (Fig. 12); malar space 0.4× as long as height of eye and 1.5× as long as basal width of mandible; frons smooth and shiny.

**Figures 13–18.**
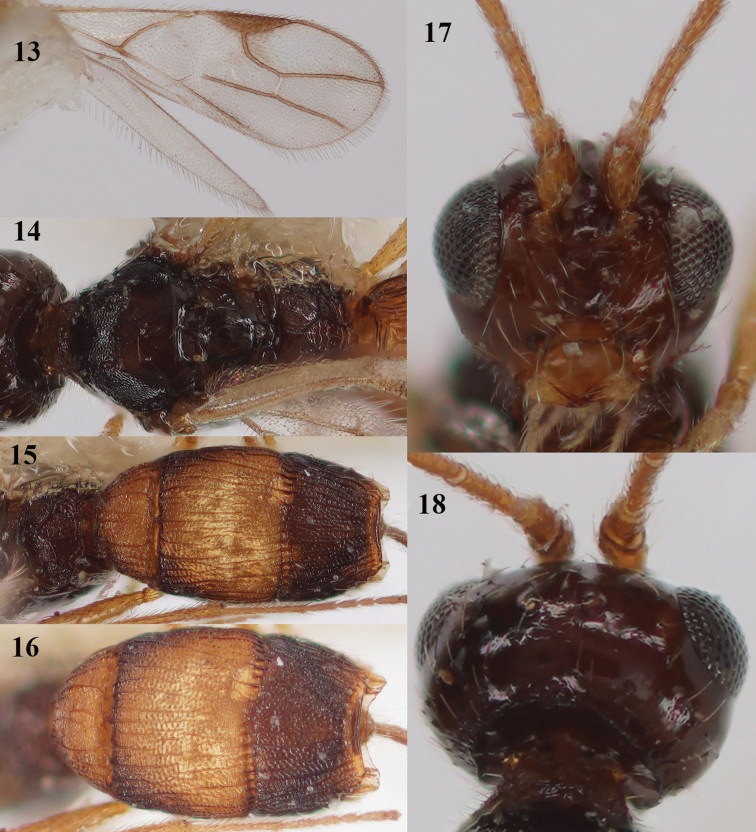
*Lysitermus tritoma* (Bouček), ♀, France **13** wings **14** mesosoma dorsal **15** propodeum and metasoma dorsal **16** second and third metasomal tergites dorsal **17** head anterior **18** head postero-dorsal. Photographs: R. Soethof.

***Mesosoma*.** Mesosoma 1.5× as long as high in lateral view; mesoscutum granulate and with 2 rather short carinulae and no medio-posterior groove (Fig. 14); notauli shallow, distinctly developed anteriorly and posterior half obsolescent, largely smooth and anteriorly connected to lateral irregular carina; scutellar sulcus with 1 carina; scutellum smooth and shiny, convex (Fig. 14); propodeum shiny, largely smooth between carinae and with large areola, its median carina approx. as long as anterior side of areola (Fig. 14); side of pronotum rugose-crenulate and with some granulate sculpture; mesopleuron largely smooth and convex (Fig. 12); precoxal sulcus rather deep and only in anterior half present, with few crenulae anteriorly; metapleuron largely smooth and with long setae; mesosternal sulcus deep, narrow and smooth. ***Wings*.** Fore wing: pterostigma elongate triangular and 3.5× longer than wide (Fig. 13), r issued from its middle and slightly longer than width of pterostigma; 2-SR completely absent (Fig. 13); 3-SR 1.5× longer than r; SR1 straight and reaching tip of wing; m-cu rather short (Fig. 13); subdiscal cell distally closed, CU1b close to m-cu and CU1a at same level as 2-CU1 (Fig. 13). ***Legs*.** Hind coxa largely smooth; length of femur, tibia and basitarsus of hind leg 5.0, 9.2, and 5.2× their width, respectively; hind tarsus hardly compressed.

***Metasoma*.** Length of first tergite 0.6× its apical width, its dorsal surface evenly convex, surface longitudinally striate and with additional granulate sculpture between striae, its dorsal carinae lamelliform and medially interconnected; medial length of second tergite 0.9× its basal width, and 1.4× as long as third tergite; second and third tergites longitudinally striate and with distinct secondary granulate sculpture; second transverse suture coarsely crenulate and nearly straight (Fig. 15); third tergite antero-laterally without minute tooth-shaped protuberance and distinctly narrowed posteriorly (Figs 15, 16), with distinct smooth and shiny apical rim (Fig. 15); apical lamella of third metasomal tergite moderately protruding laterally (Fig. 12), concave and wide in dorsal view, with approx. 8 carinae (Fig. 16); setose part of ovipositor sheath 0.21× as long as fore wing and 0.6× as long as hind tibia, slightly widened apically (Fig. 12).

***Colour*.** Dark brown; third tergite dark brown, contrasting with largely yellowish brown second tergite (Figs 15, 16); first tergite yellowish brown; antenna (basal segments yellowish), pterostigma (but basal fifth yellow) and veins M+CU1 apically, 1-CU1, 1-M, and veins of apical half of fore wing dark brown (Fig. 13); palpi and tarsi pale yellowish; remainder of legs yellowish brown; wing membrane infuscate, but band below base of pterostigma and marginal cell partly, subhyaline (Fig. 13).

**Figures 19–21.**
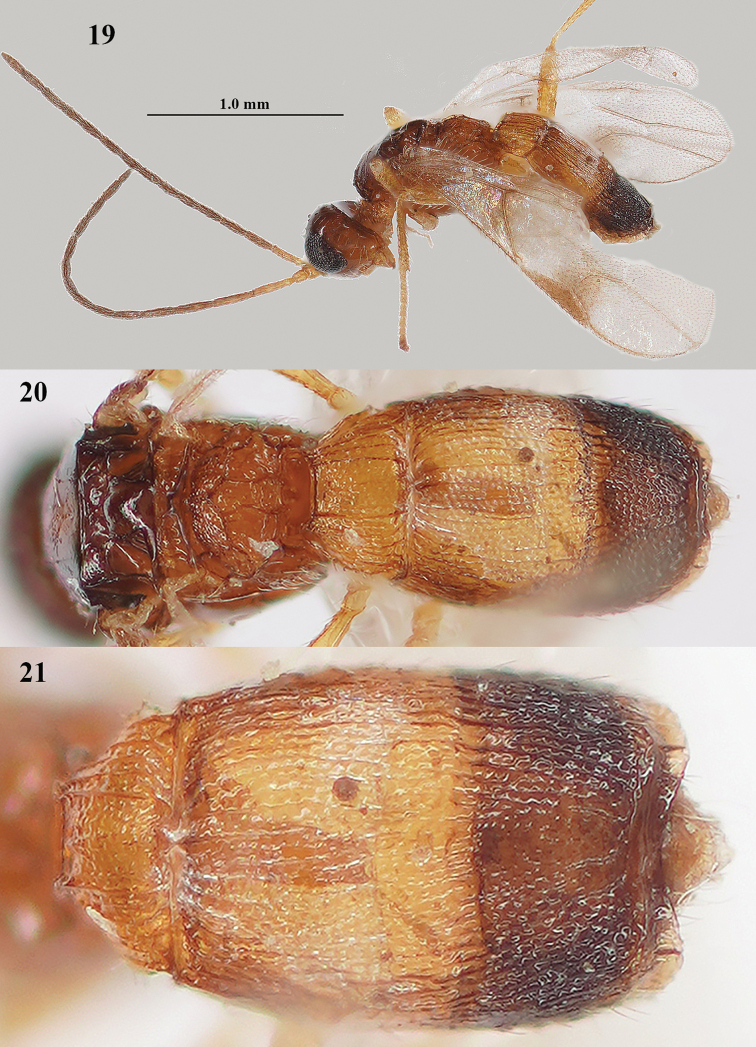
*Lysitermus tritoma* (Bouček), ♂, Malta **19** habitus dorso-lateral **20** propodeum and metasoma dorsal **21** second and third metasomal tergites dorsal. Photographs: R. Soethof.

***Variations*.** Antenna with 14–17 segments; length of body 1.5–1.9 mm, and of fore wing 1.3–1.5 mm; length of ovipositor sheath 0.21–0.26× as long as fore wing; vein 2-SR of fore wing varies from completely absent (Figs 13, 19), complete (Fig. 29) to small unsclerotised trace (Fig. 45); notauli complete to posterior third obsolescent; scutellar sulcus with 1–3 carinae; precoxal sulcus smooth or with few crenulae; metapleuron largely smooth or with some rugulae medially; median carina of propodeum approx. as long as anterior side of areola or much shorter; second metasomal tergite 1.3–1.5× longer than second tergite; lamella of third tergite hardly serrate (Fig. 12), with 8–16 carinae; colour of body is very variable: southern specimens are brownish yellow with only third tergite dark brown and northern specimens are largely dark brown (except first and second tergites, but in both Finnish specimens also darkened; Figs 27–29), sometimes hind coxa, femur and tibia largely brown or dark brown (Fig. 29).

**Male.** Very similar to female, but metasoma slenderer (Fig. 20); antenna with 16 or 17 segments; length of body 1.4–1.6 mm, and of fore wing 1.3 mm; head dark brown dorsally; linear medio-posterior depression of mesoscutum absent or slightly impressed; third tergite dark brown and contrasting with yellowish second tergite (Fig. 20), striate and/or granulate and its lamella hardly serrate (Figs 19–21, 45); pterostigma dark brown basally.

#### Biology.

Reared from lepidopterous case-bearing larvae belonging to Psychidae (*Luffia ferchaultella* (Stephens, 1850); *L. lapidella* (Goeze, 1783); *L.* sp.; *Dahlica* sp.) and Tineidae (*Eudarcia derrai* (Gaedike, 1983); Mifsud et al. 2019). It has been reared from *Inonotus radiatus* bracket fungi on *Alnus glutinosa* (L.) (Jonsell et al. 2016), most likely from host cases hiding in the fungi.

#### Distribution.

*Bulgaria, Czech Rep., *Finland, *France, Italy (Sardinia), Malta, *Netherlands, Poland, *Portugal (mainland), *Romania, *Spain (mainland), Sweden.

#### Notes.

**Figures 22–25.**
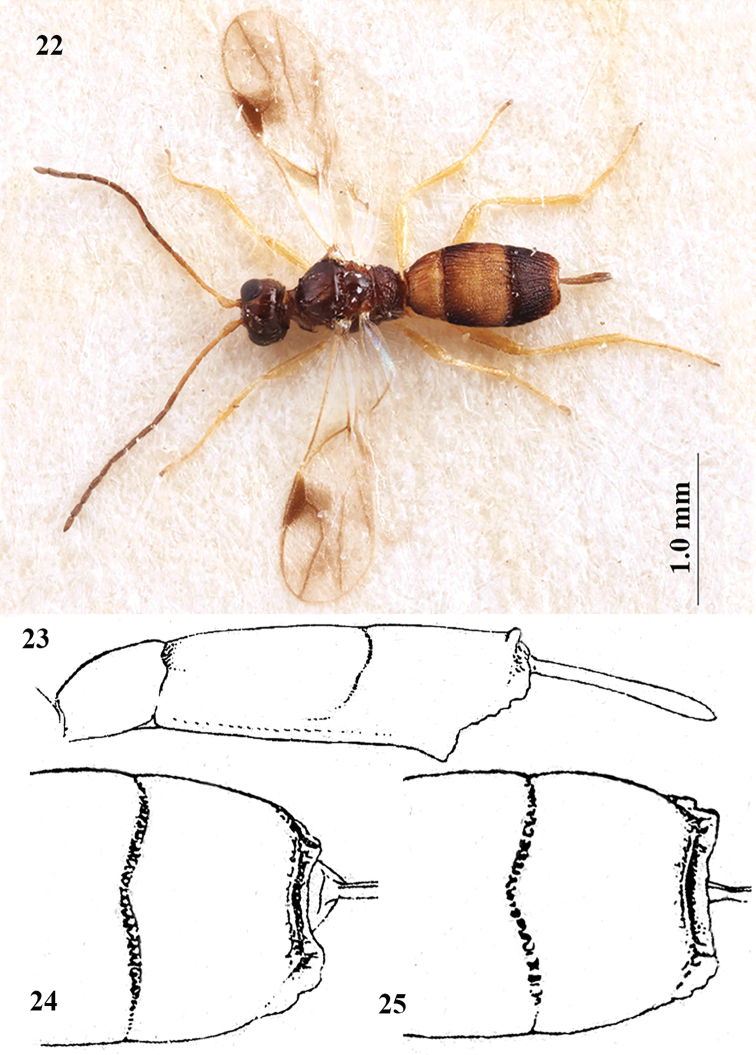
*Lysitermus tritoma* (Bouček), ♀, 22 holotype *L. suecicus*, **23–25** types of *L. tritoma***22** habitus dorsal **23** metasoma lateral **24, 25** detail third tergite dorsal. **22** from Forshage et al. (2016) and **23–25** from Bouček (1956).

The medio-longitudinal carina of the propodeum is very variable in length, from about as long as oblique anterior side of propodeal areola to much shorter and the second metasomal suture varies from distinctly sinuate (typical *L. tritoma*; Figs 24, 25) to straight (typical *L. suecicus*; Figs 15, 20, 22). After more reared specimens became available, intermediates of both characters have been found, and there are no grounds to separate any longer the two species, as has been proposed by van Achterberg (1991).

The holotype of *L. suecicus* (NRS) is incorrectly figured in the original description. For instance, the second tergite is not twice as long as the third tergite but 1.5× (Fig. 22), the ovipositor sheath is not widened but subparallel, and the pterostigma is not robust but rather slender (Fig. 22).

**Figures 26–27.**
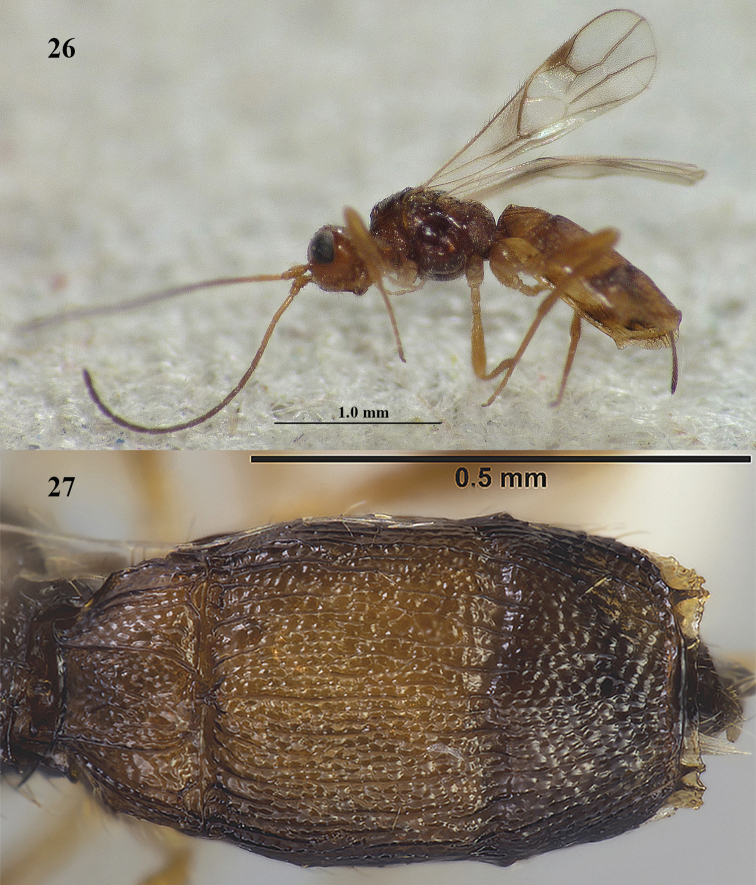
**26***Lysitermus pallidus* Foerster, ♀, Sweden (freshly emerged specimen), habitus lateral **27***L. tritoma* (Bouček), dark ♀, Finland, metasoma dorsal. Photographs: F. Skeppstedt (**26**) and P. Malinen (**27**).

**Figures 28–29.**
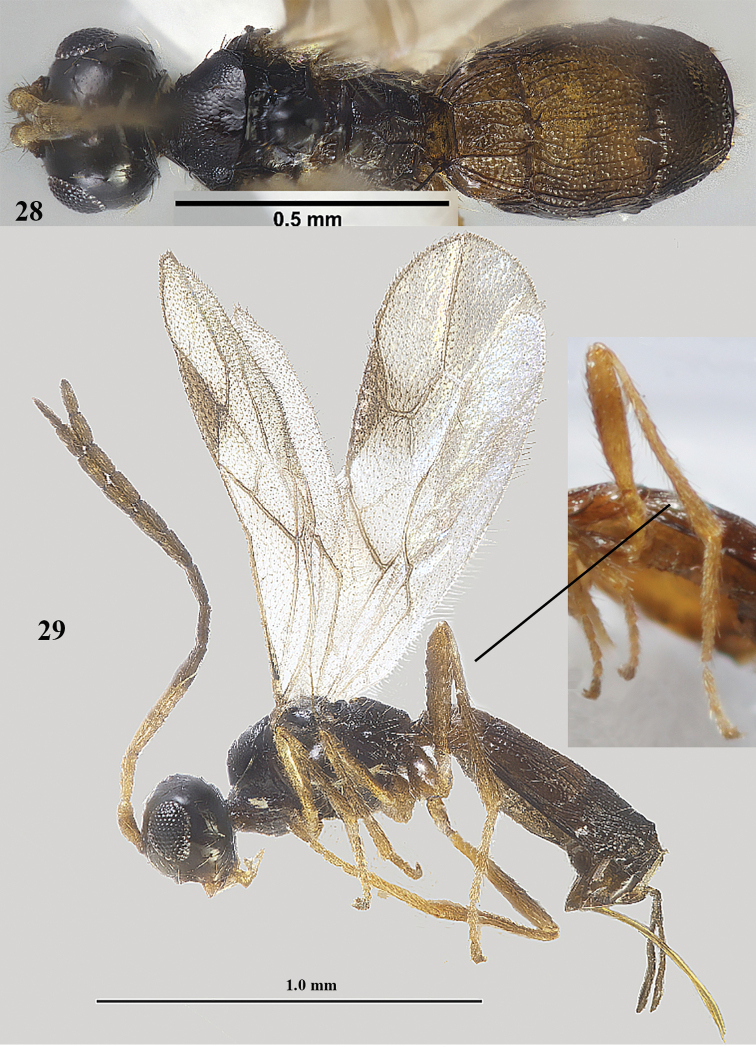
*Lysitermus tritoma* (Bouček), dark ♀, Finland **28** body dorsal **29** habitus lateral. Photograph: P. Malinen.

### Lysitermus talitzkii

Taxon classificationAnimaliaHymenopteraBraconidae

(Tobias, 1976) stat. nov.

964A0CBB-1E34-5FD1-AF31-F73F2B528CB1

Figs 30–32 , 33 , 34–42 , 43–44 , 45–47

Prolysitermus talitzkii Tobias, 1971: 205; Shenefelt 1975: 1155.
Lysitermus talitzkii ; Belokobylskij and Tobias 1986: 64 (as synonym of Lysitermus pallidus); van Achterberg 1991: 20 [examined].
Prolysitermus longiventris Tobias, 1976: 50, 253.
Lysitermus longiventris ; Belokobylskij and Tobias 1986: 64; van Achterberg 1991: 20. Syn. nov.

#### Material.

Holotype of *Prolysitermus talitzkii*, ♂ (ZISP), “MCCP [= **Moldova**], Kishinev, radiolu, 2.vi.[19]62, 65, Talitzkij”, “Holotypus *Praelysitermus* [sic!] *talitzkii* Tobias, [19]71”; 1 ♂ paratype of *P. talitzkii* (ZISP) from Moldova, Străşeni, 21.vii.1961. Holotype of *Prolysitermus longiventris*, ♂ (ZISP), “[**Russia**:] Sochi-Lazarevskoe, 3–4.v.[1]973, V. Tobias”, “*Prolysitermus longiventris* sp. n., Tobias, det. 1973”, “Holotypus *Prolysitermus longiventris* Tobias, 1976”; 1 ♀ paratype (ZISP), “28.iv.[19]75”, “[Russia:] Krasnodarskiy kray, Lazarevskoe, les na terrasirovannych sklonach, 28.iv.1975, V. Tobias”, “Paratypus *Prolysitermus longiventris* Tobias, 1976”; 1 ♂ paratype (ZISP), “11.v.[19]75”, and same label data as ♀ paratype.

#### Redescription.

Paratype ♀ of *L. longiventris*, length of fore wing 1.4 mm, and of body 1.6 mm.

**Figures 30–32.**
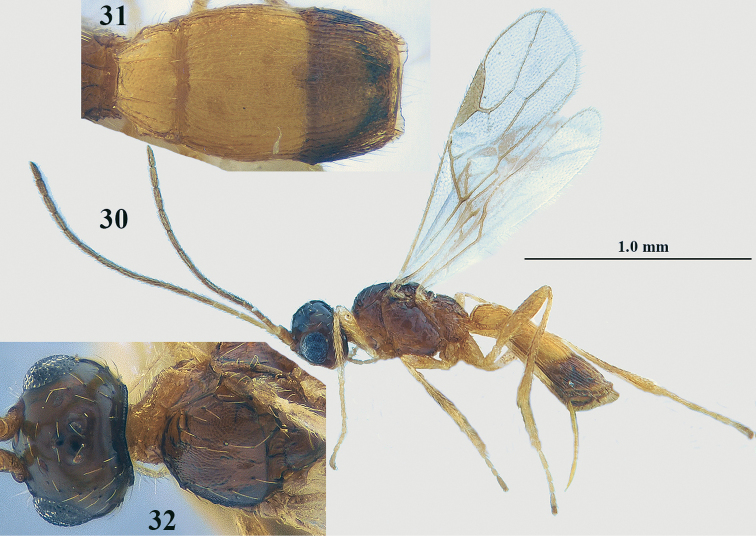
*Lysitermus talitzkii* (Tobias), ♀, paratype of *L. longiventris***30** habitus lateral **31** metasoma dorsal **32** head and mesoscutum dorsal. Photographs: K. Samartsev.

***Head*.** Antenna 1.1× as long as fore wing, with 15 segments, rather widened apically (Fig. 30), scapus oblique apically, length of third segment 1.1× fourth segment; third, fourth, and penultimate segments 6.6, 5.8, and 2.9× their width, respectively; face largely smooth, with long erect setae and 2.2× wider than high; clypeus smooth and distinctly convex; head in dorsal view 1.6× broader than long medially, eye 1.4× longer than temple dorsally, strongly and roundly narrowed posteriorly (Fig. 32); vertex smooth and shiny, including area near stemmaticum (Fig. 32); occipital carina strong medio-dorsally and in front of it micro-crenulate; POL:diameter of posterior ocellus: OOL = 20:12:35; eye in lateral view 1.5× as high as wide, temple distinctly broadening ventrally, smooth, and width of eye 1.3× minimum width of temple laterally; malar space 0.5× as long as height of eye and 1.7× as long as basal width of mandible; frons smooth and shiny.

***Mesosoma*.** Mesosoma 1.5× as long as high in lateral view; mesoscutum granulate and shiny, medio-posteriorly with indistinct groove (Fig. 32); notauli only anteriorly distinct, largely smooth, but anteriorly crenulated; scutellar sulcus with one median carina and 6 weak crenulae; scutellum largely smooth and shiny, convex (Fig. 32); propodeum shiny, largely smooth between carinae and with large areola, its median carina shorter than anterior side of areola; side of pronotum and mesopleuron largely smooth; precoxal sulcus rather shallow and only its anterior half present, largely smooth; metapleuron largely smooth and with long setae. ***Wings*.** Fore wing: pterostigma elongate triangular and 5.0× longer than wide (Fig. 30), r issued from its middle and 0.7× shorter than width of pterostigma; 2-SR absent except for some pigmentation (Fig. 30); 3-SR 3.1× longer than r; SR1 straight and reaching tip of wing; m-cu rather short and largely unpigmented; subdiscal cell distally closed, CU1b far from m-cu and CU1a above level of 2-CU1 (Fig. 30). ***Legs*.** Hind coxa largely smooth; length of femur, tibia and basitarsus of hind leg 4.5, 11.1, and 8.4× their width, respectively; hind tarsus compressed.

***Metasoma*.** Length of first tergite 0.6× its apical width, its surface longitudinally striate and with additional granulate sculpture between striae, dorsal surface evenly convex, its dorsal carinae lamelliform and medially interconnected anteriorly; medial length of second tergite 0.9× its basal width, and 1.4× as long as third tergite; second and third tergites longitudinally striate and with distinct additional granulate sculpture between striae; second transverse suture coarsely crenulate and nearly straight (Fig. 31); third tergite antero-laterally with minute tooth-shaped protuberance and gradually narrowed posteriorly (Fig. 31), with distinct and sculptured posterior rim; apical lamella of third metasomal tergite moderately protruding laterally (Fig. 31), straight and narrow medially in dorsal view, with approx. 14 carinae; setose part of ovipositor sheath 0.4× as long as hind tibia.

***Colour*.** Mainly dark brown; third tergite dark brown, contrasting with largely yellowish brown second tergite (Figs 30, 31); first tergite yellowish brown; scapus infuscated, pedicellus, third and fourth segments yellow; remainder of antenna, pterostigma, and veins M+CU1 apically, 1-CU1, 1-M, and veins of apical half of fore wing brown; palpi and tarsi pale yellowish; remainder of legs yellowish brown; wing membrane subhyaline.

**Male.** Colour very variable: body entirely dark brown (Fig. 43) to partly yellowish brown (Fig. 45); metasoma slender, darkened and antero-lateral small tooth-like protuberance of third tergite either absent (holotypes, but area slightly convex; Figs 36, 47) or present (Fig. 44); first tergite weakly narrowed posteriorly (Figs 36, 44) or parallel-sided (Fig. 47); vein 3-SR of fore wing 1.4–3.0× longer than vein r (Figs 34, 43, 45); length of body1.4–1.9 mm and fore wing 1.4–1.5 mm.

**Figure 33.**
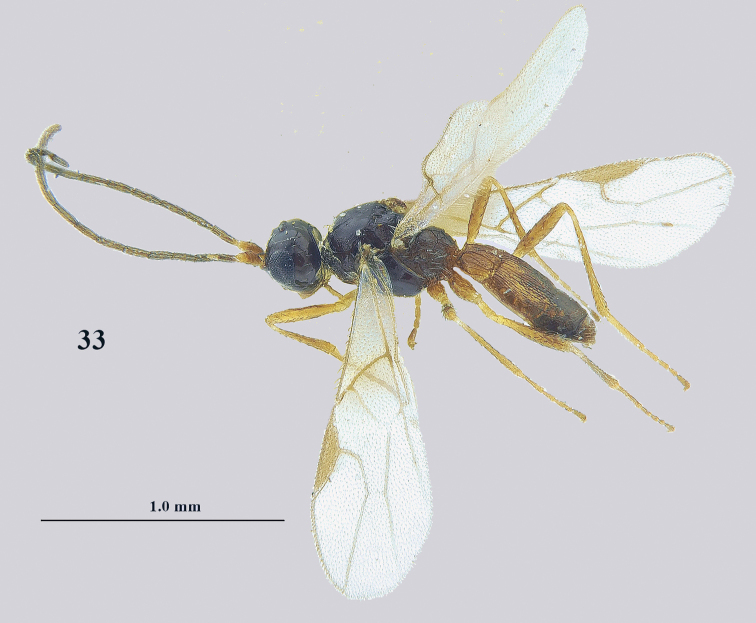
*Lysitermus talitzkii* (Tobias), ♂, holotype of *L. longiventris*, habitus lateral. Photograph: K. Samartsev.

#### Biology.

Unknown.

#### Distribution.

Moldova, Poland (Huflejt 1997), Russia.

**Figures 34–42.**
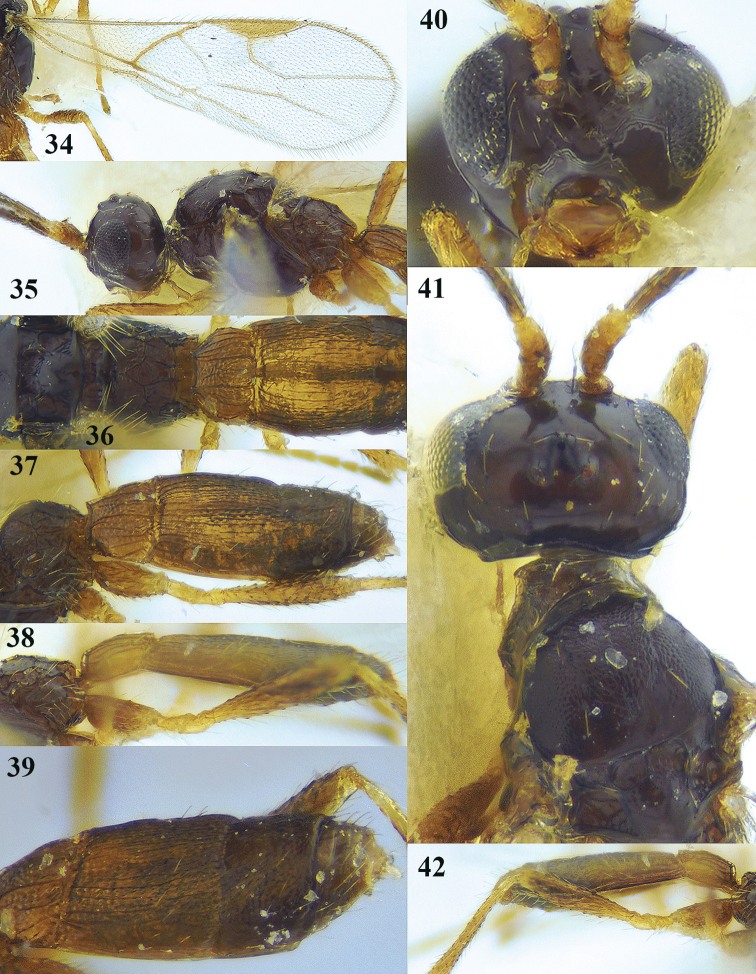
*Lysitermus talitzkii* (Tobias), ♂, holotype of *L. longiventris***34** fore wing **35** head and mesosoma lateral **36** propodeum and first and second metasomal tergites dorsal **37** metasoma dorso-lateral **38** metasoma lateral **39** metasoma dorso-lateral **40** head anterior **41** head and mesonotum dorsal **42** hind coxa and femur lateral. Photographs: K. Samartsev.

#### Notes.

The male holotype of *L. talitzkii* (Tobias, 1971) was considered to be a synonym of *L. pallidus* Foerster by Belokobylskij and Tobias (1986) and van Achterberg (1991), but after examination of reared series consisting of both sexes it is obvious that the holotype male with its gradually widened hind tibia (Fig. 45) fits better with *L. longiventris*, as defined above, and the latter is synonymised with it. The holotype has the notauli shallowly impressed posteriorly (van Achterberg 1995) although normally the posterior half of the notauli are absent, but specimens with vaguely to distinctly indicated notauli have been examined among specimens of the closely related *L. tritoma* from Sweden (holotype of *L. suecicus*), Italy, and Portugal, and, therefore, we do not consider the more developed notauli as a valid reason to maintain *L. longiventris* as a species different from *L. talitzkii*.

**Figures 43–44.**
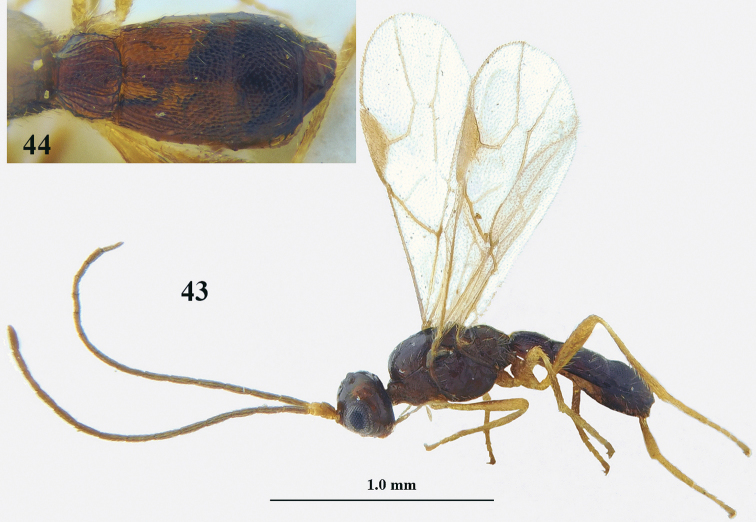
*Lysitermus talitzkii* (Tobias), ♂, paratype of *L. longiventris***43** habitus lateral **44** metasoma dorsal. Photographs: K. Samartsev.

*Lysitermus longiventris* (Tobias) was described from N. Caucasus and is very similar to *L. tritoma* (Bouček) but differs by having the third metasomal tergite often with a minute tooth-like protuberance antero-laterally and its posterior lamella straight medially or nearly so, the metasoma slightly slenderer, the third tergite slightly less narrowed posteriorly, and vein 3-SR of the fore wing usually more than twice as long as vein r. The reduction of the longitudinal rugae on the third tergite is considered less distinctive because reduction of sculpture is common in *Lysitermus* males and to a lesser degree in females. Other differences given by Belokobylskij and Tobias (1986), such as the first tergite as long as wide at apex and the second tergite longer than its apical width, disagree with the only figure in the very short original description and the figures included here (Figs 31, 36, 44). Characters such as the third tergite less sculptured than second tergite and without distinct lamella apically, the face twice as wide as high, and the width of the hypoclypeal depression equal to distance from eye to depression are more or less also found in males of *L. tritoma*.

**Figures 45–47.**
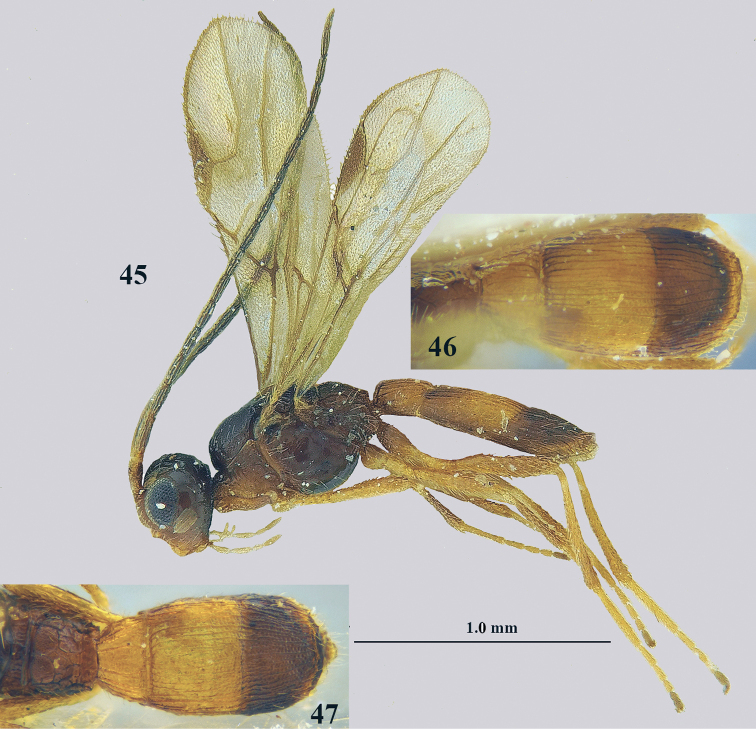
*Lysitermus talitzkii* (Tobias), ♂ holotype, but 47 of ♂ paratype **45** habitus lateral **46, 47** metasoma dorsal. Photographs: K. Samartsev.

## Supplementary Material

XML Treatment for Lysitermus

XML Treatment for Lysitermus pallidus

XML Treatment for Lysitermus tritoma

XML Treatment for Lysitermus talitzkii
